# Understanding the link between ALDH2 genotypes and diabetes

**DOI:** 10.3389/fendo.2025.1451722

**Published:** 2025-02-19

**Authors:** Miaomiao Peng, Ruikang Liu, Jiaoyue Zhang, Wen Kong, Juan Zheng, Xiang Hu, Limin Wan, Shengqing Hu, Shenghua Tian, Ying Wang, Geng Liu, Kangli Qiu, Tianshu Zeng, Lulu Chen

**Affiliations:** ^1^ Department of Endocrinology, Union Hospital, Tongji Medical College, Huazhong University of Science and Technology, Wuhan, China; ^2^ Hubei Provincial Clinical Research Center for Diabetes and Metabolic Disorders, Wuhan, China; ^3^ Department of Rehabilitation, Union Hospital, Tongji Medical College, Huazhong University of Science and Technology, Wuhan, China; ^4^ Department of Rheumatology and Immunology, Union Hospital, Tongji Medical College, Huazhong University of Science and Technology, Wuhan, China; ^5^ Department of Nuclear Medicine, Union Hospital, Tongji Medical College, Huazhong University of Science and Technology, Wuhan, China

**Keywords:** aldehyde dehydrogenase, type 2 diabetes, body mass index (BMI), waist circumference (WC), interaction effect

## Abstract

**Introduction:**

The association between aldehyde dehydrogenase-2 (ALDH2) rs671 and diabetes remains controversial, with uncertainty about whether alcohol consumption or other factors mediate or modify this relationship. This study aimed to examine the ALDH2–diabetes association using standardized clinical criteria while systematically investigating potential confounding, mediating, and interacting factors in a community-based cohort.

**Method:**

We analyzed baseline data from 4,535 participants in the China Cardiometabolic Disease and Cancer Cohort Study (4C study). Diabetes was diagnosed based on standardized clinical criteria, including fasting plasma glucose, 2-h postprandial glucose, glycosylated hemoglobin A1c (HbA1c), or documented prior diagnosis. We evaluated the association between ALDH2 rs671 and diabetes risk using both logistic and Cox regression models, with age as the time scale and adjustment for potential confounders. Comprehensive mediation and interaction analyses were performed to elucidate the underlying mechanisms.

**Result:**

Among male participants, the ALDH2 rs671 GA/AA genotype was associated with a lower diabetes risk compared to the GG genotype after adjusting for alcohol consumption and other potential confounders (OR = 0.751, 95% CI: 0.567–0.995). Subgroup analyses revealed that this protective effect was most pronounced in individuals with BMI < 24 (OR = 0.651, 95% CI: 0.448–0.947), with significant interaction *p*-values of 0.024. In mediation analysis, abdominal adiposity accounted for 30.4% (95% CI: 10.0%–127.0%) of the ALDH2–diabetes association and BMI mediated 18.9% (95% CI: 4.8%–75.4%) of this relationship, while alcohol consumption showed no significant mediating effect (*p* = 0.56).

**Conclusion:**

Our findings revealed that East Asian men with the ALDH2 GG genotype had an increased risk of diabetes compared to those with the GA/AA genotype, particularly among individuals with a BMI < 24. Interestingly, increased adiposity, especially abdominal fat, emerged as a potential mediator rather than alcohol consumption. Thus, individuals with the GG genotype, even with a relatively normal BMI, may benefit from regular moderate-intensity exercise and dietary interventions aimed at managing waist circumference.

## Introduction

Asians show increased susceptibility to metabolic disorders, particularly diabetes, compared to European populations (1). While this disparity has been attributed to genetic differences, only a limited number of specific genetic variants have been identified between East Asian and European ancestries. A landmark meta-analysis of 433,540 East Asian individuals provided robust evidence for several previously unreported diabetes-associated variants, with aldehyde dehydrogenase-2 (ALDH2) rs671 emerging as particularly noteworthy (2). This variant occurs in approximately 30%–50% of East Asians but less than 5% of European descent populations (3). The meta-analysis demonstrated that the ALDH2 rs671 G allele increases diabetes risk, suggesting a potential protective effect of the A allele (2). While the meta-analysis provided compelling evidence for this genetic association, the heterogeneous diabetes diagnostic criteria across included studies and limited adjustment for between-study confounders suggested the need for additional validation using standardized clinical measures. Studies in specific populations, such as those with coronary artery disease or obesity, have reported contradictory findings of increased diabetes risk associated with the variant (4–6), further highlighting the importance of systematic investigation in well-characterized cohorts.

ALDH2, a mitochondrial enzyme crucial for alcohol-derived acetaldehyde detoxification, shows 60%–80% reduced activity in heterozygous carriers and approximately 90% reduction in homozygous carriers of this mutation (7). Carriers typically experience alcohol sensitivity symptoms, including facial flushing, headache, and tachycardia, due to rapid acetaldehyde accumulation, leading to reduced alcohol consumption (3). While several Mendelian randomization studies have confirmed both the association between ALDH2 rs671 and alcohol consumption and the causal relationship between alcohol intake and diabetes development, evidence directly linking these factors remains limited (8–10).

Recent clinical and experimental evidence suggests that beyond its impact on alcohol consumption, the ALDH2 rs671 polymorphism significantly influences various human diseases through its role in enzymatic detoxification of lipid peroxidation-derived aldehydes and its participation in non-enzymatic metabolic processes (3). This raises important questions about whether the association between ALDH2 variants and diabetes is primarily mediated through alcohol consumption or alternative pathways. Furthermore, identifying potential modifiers of this relationship could significantly influence clinical risk assessment and prevention strategies.

Therefore, we conducted a comprehensive evaluation of the association between ALDH2 rs671 and diabetes using standardized clinical diagnostic criteria, including glycosylated hemoglobin A1c (HbA1c), fasting plasma glucose (FPG), postprandial 2-h plasma glucose (P2hPG), and prior diagnosis, while adjusting for potential confounding factors. This rigorous approach ensures reliable clinical implications and builds upon previous meta-analyses. We also investigated the extent to which alcohol consumption and other factors mediate or modify the relationship between ALDH2 rs671 polymorphism and diabetes. By elucidating the interplay between genotype and metabolic factors influencing diabetes risk, this study aims to inform the development of more effective, targeted preventive measures.

## Methods

### Subjects

This study analyzed data from the China Cardiometabolic Disease and Cancer Cohort Study (4C study), a nationwide, population-based, prospective cohort study conducted across 20 communities in mainland China. The study design has been previously detailed (11, 12). We utilized baseline survey data collected during 2011–2012 from the Yi-Ling district of Yichang City, Hubei Province. From 4,686 participants aged over 40 years who completed anthropometric measurements, questionnaire surveys, and 75-g Oral Glucose Tolerance Test (OGTT), 4,535 were included in the final analysis after excluding those with missing gender or age information, those taking anti-diabetes medications, and those with failed genotyping tests. The study protocol was approved by the Institutional Review Committee of Tongji Medical College, Huazhong University of Science and Technology (IORG No. IORG0003571) and conducted in accordance with the Declaration of Helsinki and Good Clinical Practice guidelines. All participants provided written informed consent.

### Questionnaire and physical examination

Experienced investigators conducted questionnaires (including age, gender, drinking habits, smoking habits, family history of diabetes, exercise, education, and anti-diabetes drugs) and anthropometric measurements [including height, weight, waist circumference (WC), hip circumference (HC), systolic blood pressure (SBP), and diastolic blood pressure (DBP)]. We also reviewed their medical records to verify their medical history and also collected information on diagnoses, including the time of diagnosis and hospitalizations.

Drinking status was classified as “drinker” if subjects consumed alcohol at least once a week for more than six consecutive months at any point in their lifetime and “nondrinker” otherwise. For drinkers, we documented the beverage type, frequency, and typical amount per occasion. Weekly alcohol consumption was calculated as the product of frequency and typical amount, with one Chinese unit equivalent to 22.05 g of ethanol. For nondrinkers, alcohol consumption was recorded as zero. To minimize potential confounding from beverage types (13), our final analysis included only participants who exclusively consumed liquor, as the number of individuals consuming rice wine, red wine, or beer was limited (*N* = 103).

Current smokers were defined as individuals who smoked seven or more cigarettes per week during the past 6 months, while ever smokers were those who had quit smoking within the past 6 months.

### Biochemical evaluation and genotyping assays

All participants underwent a 75-g OGTT following a minimum 10-h fast. Blood samples were collected at baseline and 2 h post-glucose load. Fasting blood samples were stored at −80°C for subsequent SNP shot assays of the rs671 genotype. Detailed protocols for OGTT, biochemical measurements, genomic DNA extraction, and SNP shot assays have been previously described (11, 12).

### Outcome definition

Diabetes was diagnosed if any of the following criteria were met: HbA1c ≥6.5%, FPG ≥7.0 mmol/L, P2hPG ≥11.1 mmol/L, or documented prior diagnosis or current use of antidiabetic medication. Homeostatic model assessment of insulin resistance (HOMA-IR) was calculated as fasting insulin concentrations (mIU/L)*FPG concentrations (mmol/L)/22.5. Homeostatic model assessment of beta-cell function (HOMA-β) was calculated as [20*fasting insulin (mU/mL)]/[FPG concentrations (mmol/L)−3.5]. Hypertension was defined as SBP ≥140 mmHg, DBP ≥90 mmHg, or prior hypertension diagnosis.

### Statistical analysis

We first assessed data distribution using the Kolmogorov–Smirnov test and the Q-Q plot. For continuous variables, we presented the mean and standard deviation (SD); for non-normal variables, we reported median and interquartile ranges. All non-normal distribution continuous variables underwent natural logarithm transformation prior to analysis. For alcohol consumption data, we added a constant 1 before logarithmic conversion to accommodate zero values.

Missing alcohol consumption data (4.3% of cases) were imputed using the expectation-maximization algorithm based on sex, age, γ-glutamyltransferase, and rs671 genotype. This single imputation approach was deemed appropriate given the low proportion of missing data (<5%). Genotype distribution was evaluated for Hardy Weinberg Equilibrium using chi-square tests.

We employed Kaplan–Meier curves to visualize diabetes-free survival across ALDH2 genotype groups, with differences assessed using log-rank tests. For multivariable analysis, we first test the proportional hazards assumption. When met, we use Cox regression with age as the time scale to analyze genotype effect on diabetes risk, adjusting for potential confounders. When violated, we considered non-proportional Cox regression models. We also constructed logistic models to estimate the OR and 95% CIs for the ALDH2 rs671–diabetes association. Linear models were used to estimate the association between genotype and continuous outcomes.

We developed three sequential adjustment models: Model 1 included age, exercise, education, smoking history, and family history of diabetes; Model 2 additionally adjusted for LDL-C, HDL-C levels, and hypertension; Model 3 further incorporated alcohol consumption. We tested for collinearity among all confounders before their inclusion in adjustment models.

To assess effect modification, we conducted subgroup analyses and calculated interaction *p*-values by introducing genotype–modifier interaction terms into logistic regression models, using likelihood ratio tests. For mediation analysis, we constructed directed acyclic graphs to visualize relationships among exposure (ALDH2 genotype), outcome (diabetes), and potential mediators (BMI, WC, HC, HOMA-IR, HOMA-β, and drink dosage) (14). We used the CMAverse R package to estimate pure natural direct effects and total natural indirect effects (15), with 95% CIs obtained through nonparametric bootstrapping.

To evaluate result robustness, we performed three sensitivity analyses: (1) Cox proportional hazard modeling with age as the time scale, (2) E-value calculations to assess unmeasured confounding in mediation analyses (16), and (3) complete case analysis without imputed alcohol data.

All statistical analyses were performed using SPSS 26.0 (IBM, USA) and R statistical software version 4.3.2 (R Foundation for Statistical Computing, Austria) with the CMAverse package. All *p*-values were two-sided, with statistical significance set at *p* ≤ 0.05.

## Results

### Characteristics of study participants

Our analysis included 2,272 men and 2,263 women. The ALDH2 rs671 genotype distribution showed 3,160 participants with the GG genotype, 1,249 (27.5%) with the GA genotype, and 126 (3.0%) with the AA genotype, yielding an A allele frequency of 16.5%. This distribution conformed to Hardy–Weinberg equilibrium (*p* = 0.981), and the frequency of A allele carriers (GA/AA, 30.5%) aligned with previously reported prevalence in East Asian populations (3). Missing data comprised less than 5% of total observations. Combined outcomes of both genders are shown in **Supplementary Table S1**.

As depicted in **Table 1**, participants with the GG genotype demonstrated significantly higher alcohol consumption and prevalence of alcohol use compared to GA/AA carriers across both genders (*p* < 0.001). Among male participants, GG carriers showed higher diabetes prevalence (18.7% vs. 14.1%, *p* = 0.008) and demonstrated significantly higher level in BMI (23.35 vs. 22.71 kg/m^2^), FPG (5.78 vs. 5.67 mmol/L), P2hPG (6.75 vs. 6.48 mmol/L), and HOMA-IR (1.27 vs. 1.1) (all *p* < 0.001). Other characteristics, including education level, family history of diabetes, physical activity, and smoking behavior, were comparable between genotype groups. In women, only smoking status differed significantly between genotypes beyond alcohol consumption patterns. Based on these findings, subsequent analyses focused on the male population.

**Table 1:** Baseline characteristics of study participants stratified by sex and ALDH2 genotype.

Outcomes	Male (*N* = 2,272)			Female (*N* = 2,263)		
	GG (*N* = 1,597)	GA/AA (*N* = 675)	*p*-value^h^	GG (*N* = 1,563)	GA/AA (*N* = 700)	*p*-value^h^
Age^d^ (year)	55.38 ± 8.72	56.17 ± 8.77	0.078	54.21 ± 8.69	53.96 ± 8.8	0.527
BMI^d^ (kg/m^2^)	23.35 ± 3.14	22.71 ± 3.06	<0.001*	23.43 ± 3.25	23.44 ± 3.26	0.910
Education (uneducated/primary/junior/senior and above)	571/679/250/52	265/278/105/13	0.102	851/488/160/19	397/216/65/7	0.628
Family history of diabetes^e^ (*n*, %)	71 (4.4)	27 (4.0)	0.635	77 (4.9)	30 (4.3)	0.507
Physical activity^a,e^ (*n*, %)	344 (27.4)	143 (27.1)	0.880	326 (26.2)	147 (26.4)	0.932
Smoker^c,e^ (*n*, %)	472 (31.3)	206 (31.7)	0.864	76 (5.2)	18 (2.7)	0.010*
Drinker^b,e^ (*n*, %)	1175 (78.1)	324 (50.2)	<0.001*	307 (20.4)	37 (5.4)	<0.001*
Drink dosage^f^ (g/d)	44 (6.29–88)	1.57 (0–44)	<0.001*	0 (0–0)	0 (0–0)	/
Natural logarithm of drink dosage^g^	3.10 ± 1.82	1.85 ± 1.96	<0.001*	0.61 ± 1.30	0.16 ± 0.71	<0.001*
γ-glutamyltransferase (U/L)^f^	30 (20–50)	23 (17–34.25)	<0.001*	17 (13–26)	16 (13–24)	0.089
Natural logarithm of γ-glutamyltransferase	3.54 ± 0.76	3.25 ± 0.62	<0.001*	2.96 ± 0.53	2.91 ± 0.53	0.075
Diabetes^e^ (*n*, %)	298 (18.7)	95 (14.1)	0.008*	240 (15.4)	87 (12.4)	0.067
FPG^f^ (mmol/L)	5.78 (5.35–6.27)	5.67 (5.26–6.1)	<0.001*	5.7 (5.3–6.2)	5.7 (5.3–6.1)	/
Natural logarithm of FPG^g^	1.78 ± 0.20	1.75 ± 0.18	<0.001*	1.77 ± 0.17	1.76 ± 0.17	0.521
P2hPG^f^ (mmol/L)	6.75 (5.54–8.675)	6.48 (5.40–7.76)	<0.001*	7.1 (6.0–8.6)	6.9 (5.9–8.4)	/
Natural logarithm of P2hPG^g^	1.96 ± 0.40	1.91 ± 0.38	<0.001*	2.00 ± 0.33	1.98 ± 0.32	0.147
HbA1c^f^ (%)	5.5 (5.3–5.8)	5.6 (5.3–5.8)	0.816	5.5 (5.2–5.9)	5.6 (5.3–5.9)	/
Natural logarithm of HbA1c^g^	1.73 ± 0.13	1.73 ± 0.12	0.858	1.72 ± 0.13	1.72 ± 0.11	0.955
HOMA-IR^f^	1.27 (0.83–1.96)	1.1 (0.75–1.7)	<0.001*	1.59 (1.16–2.27)	1.64 (1.17–2.39)	/
Natural logarithm of HOMA-IR^g^	0.24 ± 0.66	0.14 ± 0.69	0.001*	0.50 ± 0.58	0.53 ± 0.57	0.340
HOMA-β^f^	41.52 (27.76–60.97)	40.87 (28.81–58.97)	0.759	55.53 (39.82–80.04)	58.61 (42.41–83.61)	/
Natural logarithm of HOMA-β^g^	3.72 ± 0.64	3.72 ± 0.60	0.889	4.02 ± 0.57	4.06 ± 0.57	0.096
Hypertension (*n*, %)	796 (49.8)	306 (45.3)	0.049*	698 (44.7)	324 (46.3)	0.472
LDL-C^d^ (mmol/L)	2.93 ± 0.85	2.87 ± 0.80	0.159	3.03 ± 0.82	3.07 ± 0.83	0.350
HDL-C^d^ (mmol/L)	1.73 ± 0.50	1.69 ± 0.45	0.140	1.71 ± 0.38	1.70 ± 0.36	0.444

BMI, body mass index; T2DM, type 2 diabetes mellitus; FPG, fasting plasma glucose; P2hPG, postprandial 2 h plasma glucose; HbA1c, glycosylated hemoglobin A1c; HOMA-IR, Homeostatic model assessment of insulin resistance; HOMA-β, Homeostatic model assessment of beta-cell function; LDL-C, low-density lipoprotein cholesterol; HDL-C, high-density lipoprotein cholesterol. ^a^Physical activity was defined as “Yes” when leisure-time physical activity exceeded 30 min per day within the past 7 days and was otherwise defined as “No”, based on Physical Activity Guidelines Advisory Committee Report, 2023 by the US Department of Health and Human Services, available at: https://health.gov/sites/default/files/2019-09/Physical_Activity_Guidelines_2nd_edition.pdf. ^b^Drinkers were defined as subjects who drank at least once a week for more than 6 consecutive months at any point in their lifetime. ^c^Smokers were defined as individuals who smoked during the past 6 months. ^d^Normally distributed variables are presented as the mean ± standard deviation. ^e^Categorical variables are presented as numbers (percentages). ^f^Non-normally distributed variables are presented as the median (interquartile range). ^g^Non-normally distributed variables were naturally log-transformed. ^h^ *p*-values are from χ^2^ test for categorical variables, from Student’s *t*-test for normally distributed variable, and Mann–Whitney *U* test for non-normally distributed variables. *p ≤ 0.05.

### ALDH2 rs671 GA/AA genotype and decreased diabetes risk

After adjusting for potential confounders including alcohol consumption, male GA/AA carriers showed approximately 30% lower diabetes risk compared to GG carriers (OR = 0.751, 95% CI: 0.567–0.995) and 43.79% lower insulin resistance (HOMA-IR). This association was attenuated after further adjustment for BMI and WC. Similar relationships were observed with FPG and P2hPG levels, while HbA1c associations did not reach statistical significance (see **Table 2**).

**Table 2:** Multivariable analyses of associations between the ALDH2 rs671 genotype and diabetes-related outcomes in male participants.

GA/AA Vs. GG	Original model		Adjusting model 1^a^		Adjusting model 2^b^		Adjusting model 3^c^		Adjusting model 4^d^	
	OR/MD^f^ [95% CI]	*p*	OR/MD^f^ [95% CI]	*p*	OR/MD^f^ [95% CI]	*p*	OR/MD^f^ [95% CI]	*p*	OR/MD^f^ [95% CI]	*p*
Multiple linear/logistic regression										
T2DM	0.714 [0.556, 0.918]	0.008*	0.715 [0.551, 0.927]	0.011*	0.741 [0.570, 0.962]	0.025*	0.751 [0.567, 0.995]	0.046*	0.785 [0.602, 1.023]	0.073
FPG^e^	−1.90% [−2.91%, −0.90%]	<0.001*	−1.85% [−2.85%, −0.85%]	<0.001*	−1.81% [−2.83%, −0.79%]	<0.001*	−1.41% [−2.43%, −0.40%]	0.005*	−1.06% [−2.07%, −0.05%]	0.032*
P2hPG^e^	−3.30% [−5.08%, −1.52%]	<0.001*	−3.25% [−5.03%, −1.47%]	<0.001*	−3.15% [−4.93%, −1.37%]	0.001*	−2.29% [−4.28%, −0.30%]	0.021*	−1.37% [−3.25%, 0.51%]	0.156
HbA1c^e^	−0.01% [−0.75%, 0.73%]	0.864	0% [−0.69%, 0.69%]	0.972	0.06% [−0.64%, 0.75%]	0.910	−0.06% [−0.75%, 0.64%]	0.896	0.12% [−0.58%, 0.81%]	0.718
HOMA−IR^e^	−41.73% [−65.75%, −17.72%]	0.001*	−41.03% [−64.30%, −17.76%]	0.001*	−42.60% [−64.30%, −20.91%]	<0.001*	−43.79% [−67.06%, −20.51%]	<0.001*	−14.99% [−34.32%, 4.34%]	0.136
HOMA−β^e^	0.11% [−1.42%, 1.64%]	0.889	0.22% [−1.26%, 1.69%]	0.779	−0.03% [−1.45%, 1.40%]	0.984	−0.51% [−2.04%, 1.02%]	0.508	1.08% [−0.30%, 2.45%]	0.124
BMI	−0.599 [−0.887, −0.311]	<0.001*	−0.542 [−0.826, −0.258]	<0.001*	−0.564 [−0.819, −0.309]	<0.001*	−0.325 [−0.597, −0.053]	0.020*	/	/
Waist circumference	−2.44 [−3.287, −1.593]	<0.001*	−2.363 [−3.2, −1.526]	<0.001*	−2.418 [−3.161, −1.675]	<0.001*	−1.565 [−2.357, −0.773]	<0.001*	/	/
Hip circumference	−1.06 [−1.642, −0.478]	<0.001*	−1.012 [−1.596, −0.428]	0.001	−1.03 [−1.579, −0.481]	<0.001*	−0.852 [−1.444, −0.26]	0.005*	/	/
Multivariate Cox regression ^g^										
T2DM	0.690 [0.548, 0.870]	0.002*	0.725 [0.571, 0.920]	0.008*	0.740 [0.582, 0.941]	0.014*	0.747 [0.581, 0.960]	0.023*	0.815 [0.639, 1.039]	0.099

BMI, body mass index; OR, odds ratio; MD, mean difference; CI, confidence interval. ^a^Model 1 includes age, smoking status, education, physical activity, and family history of diabetes. ^b^Model 2 = Model 1+LDL-C+HDL-C+hypertension. ^c^Model 3 (full model) = Model 2+drink dosage. ^d^Model 4 (sensitivity model) = Model 2+BMI+waist circumference. ^e^Non-normally distributed variables were natural log transformed and resulted in percentage differences. ^f^GG was regarded as control group in the regression analyses. ^g^Age is regarded as the time variable in the Cox regression, and the adjusting models are the same as linear/logistic regression (only age is excluded from the adjusting model). *p ≤ 0.05.

**Figure 1:**
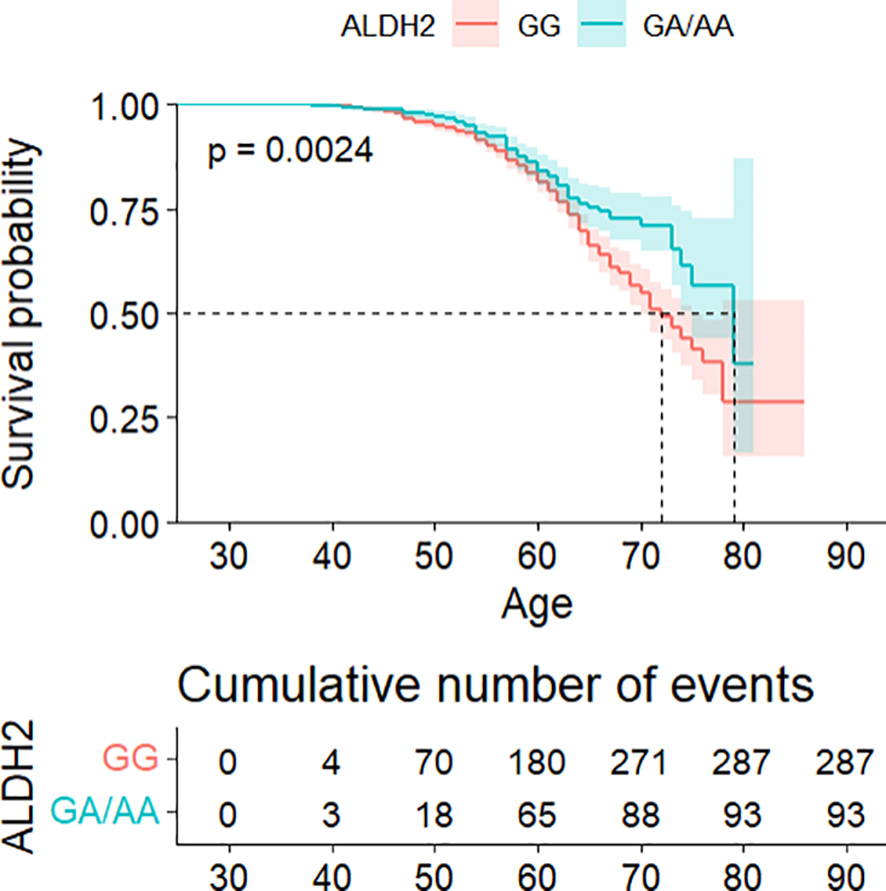
Kaplan–Meier analysis of cumulative diabetes risk stratified by ALDH2 genotype, with age as the primary time scale.

Survival analysis demonstrated significantly delayed diabetes onset in GA/AA carriers compared to GG carriers (*p* = 0.0024) (see **Figure 1**). Multivariate Cox regression confirmed this association (HR = 0.747, 95% CI: 0.581–0.960) after controlling for confounders, though the difference became non-significant following adjustment for BMI and WC (see **Table 2**). The association between the ALDH2 rs671 A allele count and diabetes risk consistently supported these findings (see **Supplementary Table S4**).

### Body mass index and waist circumference modify ALDH2–diabetes association

Subgroup analyses revealed significant interaction effects exclusively between ALDH2 genotype and anthropometric measures. Both BMI (*p* = 0.037) and WC (*p* = 0.024) modified the association between the ALDH2 genotype and diabetes risk. No significant interactions were observed with other factors including age, exercise habits, education level, smoking status, alcohol consumption, hypertension, or LDL-C levels (see **Table 3**).

**Table 3:** Stratified analyses examining the association between the ALDH2 genotype and diabetes risk across different population subgroups in male participants.

Characteristics	Subgroups	Total	Event (*n*, %)		Adjusted OR^e^	Adjusted *p*-value for interaction^d^
			GG	GA/AA		
Age (years)	<50	656	64 (13.4)	15 (8.4)	0.507 [0.262, 0.983]	0.531
	≥50, <60	845	109 (18.4)	46 (18.3)	1.214 [0.782, 1.886]	
	≥60	771	125 (23.8)	34 (13.9)	0.549 [0.344, 0.874]	
BMI (kg/m^2^)	<24	1,406	163 (17.1)	48 (10.5)	0.651 [0.448, 0.947]	0.024*
	≥24	837	131 (21.0)	46 (21.7)	1.003 [0.650, 1.547]	
Waist circumference (cm)	<85	1,588	181 (16.9)	57 (11.0)	0.696 [0.492, 0.984]	0.035*
	≥85	684	117 (22.1)	38 (24.3)	1.115 [0.699, 1.780]	
Physical exercise^a^	Active	1,295	174 (19.1)	51 (13.2)	0.663 [0.475, 0.926]	0.242
	Inactive	487	57 (16.6)	23 (16.1)	1.038 [0.613, 1.757]	
Education	Uneducated	836	114 (20.0)	32 (12.1)	0.665 [0.417, 1.058]	0.166
	Primary	957	118 (17.4)	38 (13.7)	0.762 [0.491, 1.183]	
	Junior	355	45 (18.0)	18 (17.1)	0.813 [0.411, 1.606]	
	Senior and above	65	10 (19.2)	4 (30.8)	1.649 [0.198, 13.751]	
Smoker^b^	No	653	93 (20.3)	31 (15.9)	0.721 [0.437, 1.189]	0.767
	Current smoker	678	81 (17.2)	24 (11.7)	0.777 [0.457, 1.321]	
	Ever smoker	826	102 (17.7)	36 (14.5)	0.776 [0.492, 1.224]	
Drinker	No	651	52 (15.8)	48 (14.9)	0.891 [0.567, 1.402]	0.299
	Yes	1,499	231 (19.7)	42 (13.0)	0.640 [0.442, 0.925]	
Hypertension	No	1,170	126 (15.7)	37 (10.0)	0.618 [0.401, 0.952]	0.307
	Yes	1,102	172 (21.6)	58 (19.0)	0.863 [0.592, 1.256]	
LDL-C (mmol/L)^c^	<3.4	1,675	204 (17.6)	66 (12.8)	0.686 [0.488, 0.965]	0.545
	≥3.4	592	94 (21.8)	29 (18.1)	0.890 [0.535, 1.483]	

BMI, body mass index; LDL-C, low-density lipoprotein cholesterol. ^a^Physical activity was defined as “Yes” when leisure-time physical activity exceeded 30 min per day within the past 7 days and was otherwise defined as “No”, based on Physical Activity Guidelines Advisory Committee Report, available at: https://health.gov/sites/default/files/2019-09/Physical_Activity_Guidelines_2nd_edition.pdf. ^b^Smoker (ever yes) was defined as individuals who smoked before but quit smoking during the past 6 months. ^c^Standard of subgroup separation is referred to Chinese guidelines for lipid management (2023). ^d^ *p*-value was adjusted by age, education, exercise, smoking status, family history of diabetes, hypertension, LDL-C, HDL-C, and drink dosage. Targeting variable of data analysis would not be included in adjusting model. ^e^GG was regarded as control group in the logistic regression analysis. *p ≤ 0.05.

### Abdominal adiposity mediated ALDH2–diabetes association

**Figure 2:**
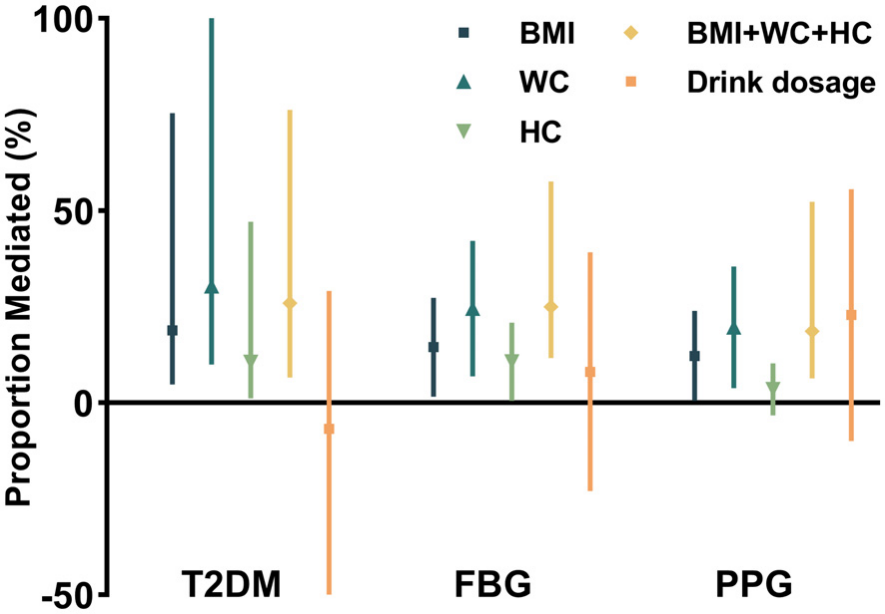
Proportional mediation effects of anthropometric and metabolic factors on the association between ALDH2 genotype and diabetes risk.

Mediation analysis, adjusted for potential confounders, revealed that anthropometric measures partially mediated the association between ALDH2 rs671 and diabetes risk. WC showed the strongest mediating effect, accounting for 30.4% (95% CI: 10.0%–127.0%) of the association, followed by BMI at 18.9% (95% CI: 4.8%–75.4%) and HC at 10.6% (95% CI: 1.2%–47.1%). The combined effect of all significant mediators was 26.0% (95% CI: 6.6%–76.2%). Notably, alcohol consumption showed no significant mediating effect (*p* = 0.56) (see **Figure 2**). Additional analyses revealed that insulin resistance (HOMA-IR), but not beta-cell function (HOMA-β), significantly mediated the ALDH2–diabetes relationship. However, this mediating effect of HOMA-IR was attenuated after accounting for WC, suggesting that increased abdominal adiposity and subsequent insulin resistance play key roles in this association (see **Supplementary Table S5**). These findings remained robust in sensitivity analyses using the complete dataset without imputed alcohol consumption data (see **Supplementary Table S2**). The more specific estimates of mediation analysis parameters and the sensitivity analysis for unmeasured confounding are also included in **Supplementary Table S2**.

## Discussion

In this community-based study, male carriers of the ALDH2 rs671 GG genotype demonstrated increased diabetes susceptibility compared to GA/AA carriers. This association persisted after adjusting for sociodemographic and lifestyle factors, including alcohol consumption, and was particularly pronounced in individuals with BMI < 24. Notably, alcohol consumption neither modified nor mediated the ALDH2–diabetes association. Instead, WC emerged as a significant mediator, accounting for approximately 30% of this relationship. These findings suggest that individuals with the GG genotype, especially those with normal BMI, should monitor their glucose levels carefully. Importantly, the elevated risk associated with this unmodifiable genetic factor can be mitigated through lifestyle interventions targeting WC reduction (17).

Our observation of a nearly 30% lower diabetes risk in male GA/AA carriers compared to GG carriers aligns with previous findings in Japanese populations (18). The protective effect extended to other diabetes-related traits in that GG carriers demonstrated significantly higher levels of FPG, P2hPG, and HOMA-IR (all *p* < 0.001). The absence of significant differences in HbA1c likely reflects its nature as a more specific but less sensitive indicator of glucose metabolism dysregulation compared to FPG and P2hPG (19).

Similar to previous studies, the ALDH2–diabetes association was only significant in male participants (2, 20). The sex-specific effects observed in our study can be explained by our mediation analysis findings that abdominal adiposity significantly mediates the ALDH2–diabetes relationship. Male participants exhibit greater susceptibility to visceral fat accumulation due to the absence of estrogen's protective effect (21), and this sex-specific pattern of fat distribution may amplify ALDH2's impact on visceral adiposity in men. This hypothesis is supported by Wang et al.'s findings that ALDH2 variants significantly influenced fat distribution specifically in male participants (22). Furthermore, experimental evidence suggests that estrogen may directly modulate ALDH2 activity (23), providing additional insight into the sex-specific effects.

Our findings align with the large-scale meta-analysis by Spracklen et al. (2), where they reported that the ALDH2 Glu504Lys G allele (encoding normal enzyme activity) was associated with increased T2D risk (OR = 1.17). While they focused on reporting the effect of the major G allele, our study examined the protective effect of the minor A allele, essentially describing the same genetic association from complementary perspectives.

Comprehensive subgroup analyses confirmed the robustness of our findings. The association between ALDH2 and diabetes risk remained consistent across various risk strata, including age, physical activity, education, and smoking behavior. In line with previous studies by Husemoen et al. and Li et al., although the diabetes-protective effects of the A allele appeared more pronounced in drinkers, we found no significant interaction between drinking behavior and ALDH2 genotype regarding diabetes risk (24, 25).

Notably, the protective effect of the A allele was most evident in participants with BMI < 24, which helps reconcile seemingly contradictory findings in previous literature. Our previous meta-analysis of 46 studies with over 90,000 participants revealed heterogeneous associations between ALDH2 variants and diabetes risk (26). The present study suggests that BMI may be a key effect modifier, as studies demonstrating the protective effects of the A allele were predominantly conducted in non-obese populations (18, 27), while those reporting null or opposite associations focused on overweight populations (4, 5).

This BMI-dependent effect likely stems from obesity-induced oxidative stress. Excessive adipose tissue accumulation leads to increased generation of 4-hydroxynonenal (4-HNE), a reactive aldehyde derived from lipid peroxidation (28, 29). 4-HNE induces cytotoxicity, glutathione depletion, and mitochondrial dysfunction, ultimately contributing to obesity-associated insulin resistance (30). As ALDH2 is the primary enzyme responsible for 4-HNE detoxification (3), obese individuals carrying the less active A allele may experience metabolic deterioration due to the mismatch between elevated 4-HNE production and reduced detoxification capacity, thus diminishing the otherwise protective metabolic effects of this variant.

**Figure 3:**
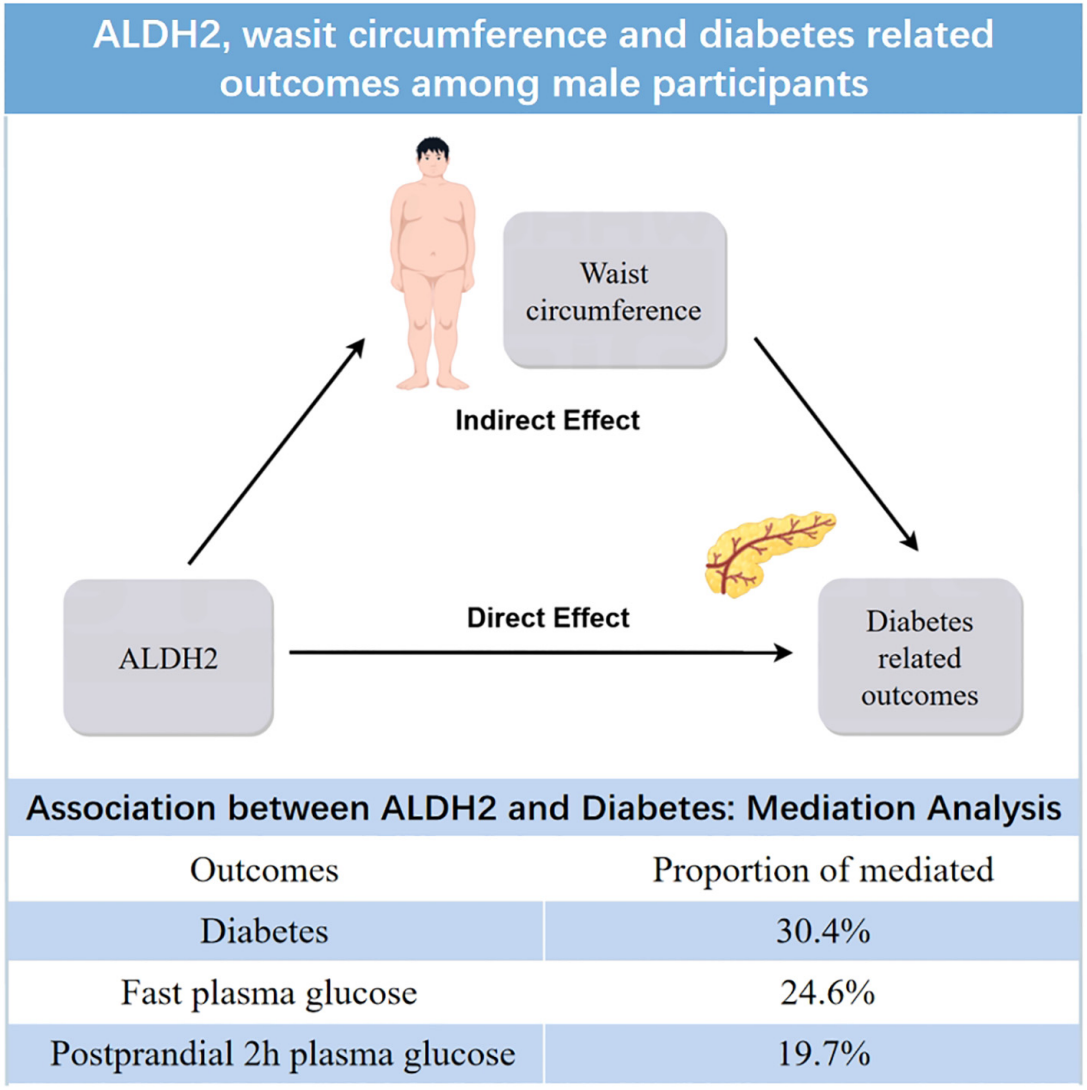
Waist circumference mediates the relationship between ALDH2 and diabetes related outcomes.

The exact mechanism linking ALDH2 rs671 polymorphism to diabetes risk has been debated. While previous studies suggested that altered drinking behavior might mediate this relationship due to ALDH2's well-established role in alcohol metabolism, our mediation analysis revealed that alcohol consumption had a limited and statistically non-significant mediating effect. These findings remained robust in sensitivity analyses using complete data without imputed alcohol values (**Supplementary Tables S2**, **S3**). Instead, our analysis revealed that approximately 30% of the diabetes risk reduction could be attributed to the effect of ALDH2 rs671 on WC (see **Figure 3**).

Molecular studies have identified ALDH2's direct biological role in adipocyte differentiation and adipogenesis (31), which may represent the potential mechanism through which ALDH2 influences diabetes risk by regulating both fat accumulation (BMI) and ectopic fat distribution (WC). These findings align with evidence from the Fat Distribution and Disease (FADE) cohort, where Wang et al. utilized MRI to assess visceral fat area (VFA) and subcutaneous fat area (SFA), demonstrating that the ALDH2 rs671 G allele was specifically associated with increased visceral fat accumulation (22), which is a well-established risk factor for insulin resistance and type 2 diabetes. While these molecular and imaging findings support the mediating effects observed in our analysis, the pathways accounting for the remaining mediation effects remain to be elucidated. ALDH2 may also influence body composition through behavioral pathways, including dietary preferences (32, 33). Importantly, our findings demonstrate that regardless of the underlying mechanisms, the diabetes risk associated with ALDH2 variants can be effectively mitigated through lifestyle modifications, particularly weight management and WC control.

The present study has several notable strengths. It utilized a relatively large community population encompassing individuals with diverse health backgrounds, rather than focusing on a specific disease cohort as in previous studies, thereby minimizing selection bias. Secondly, the study employed standardized clinical diagnostic criteria for diabetes, enhancing its clinical relevance. Thirdly, while the sample size was smaller than previous meta-analyses, our study offered the advantage of consistent methodology and measurements. The analysis of individual-level data ensured uniform methodology and avoided common study-level confounders encountered in meta-regression analysis (34). Furthermore, we explicitly defined the roles of covariates as either interactors or mediators and conducted mediation analysis within a counternatural framework, enabling comparison of relative mediator importance.

Despite these strengths, several limitations warrant consideration. Firstly, as with any observational study, there may be residual confounding despite our efforts to adjust for potential confounding factors. Although our findings of ALDH2's effects on diabetes risk through adiposity measures are supported by molecular studies and imaging evidence, future research would be valuable to further elucidate the underlying mechanisms. Specifically, studies investigating the molecular pathways linking ALDH2 to adipose tissue distribution, potential behavioral mediators, and the interaction between ALDH2 variants and lifestyle interventions could provide additional insights for developing more targeted preventive strategies.

Secondly, although type 1 diabetes and maturity onset diabetes of the young (MODY) are relatively rare compared forms of diabetes, the absence of diabetes-related antibody and MODY gene testing may have led to their inadvertent inclusion in our study population. Additionally, our reliance on questionnaire responses for alcohol consumption data introduces potential recall bias. However, we were able to partially validate this self-reported drinking information through the confirmation of well-established associations between alcohol consumption, ALDH2 rs671 genotype, and γ-glutamyltransferase levels in our dataset (see **Supplementary Table S3**, **Supplementary Figure S2**). Finally, while the mediation analysis assumed a specific sequence of effects, the cross-sectional design limited our ability to establish causality definitively—a common limitation in cross-sectional studies.

## Conclusion

Our studies show that the ALDH2 GG genotype is linked to an elevated risk of diabetes in male participants compared to the GA/AA genotype, possibly due to their increased adiposity, especially abdominal fat, rather than alcohol consumption, with this association being particularly notable in individuals with BMI < 24. These results may indicate that genetic predisposition to diabetes risk can be modified through WC control, even in those with normal BMI, suggesting the importance of targeted lifestyle interventions such as regular moderate-intensity exercise and dietary modifications among metabolically susceptible East Asian populations where this variant is prevalent. Future research should focus on elucidating the molecular mechanisms underlying this relationship and evaluating the effectiveness of targeted interventions in different ALDH2 genotype groups. Our findings highlight the potential value of considering ALDH2 genotype status when developing personalized diabetes prevention strategies, particularly in East Asian populations.

## Data Availability

The datasets presented in this study can be found in online repositories. The names of the repository/repositories and accession number(s) can be found in the article/**Supplementary Material**.

## Supplementary material

Supplementary Figure 1 The DAG depicting the relationship between confounders, exposure, mediators and outcome.

Supplementary Figure 2 Association between γ-glutamyltransferase and drinking dosage.
