# Supplementary figures and images for: Understanding the link between ALDH2 genotypes and diabetes
Source: Front Endocrinol (Lausanne). 2025 Feb 19;16:1451722. doi: 10.3389/fendo.2025.1451722 (PMC11879816; doi:10.3389/fendo.2025.1451722)

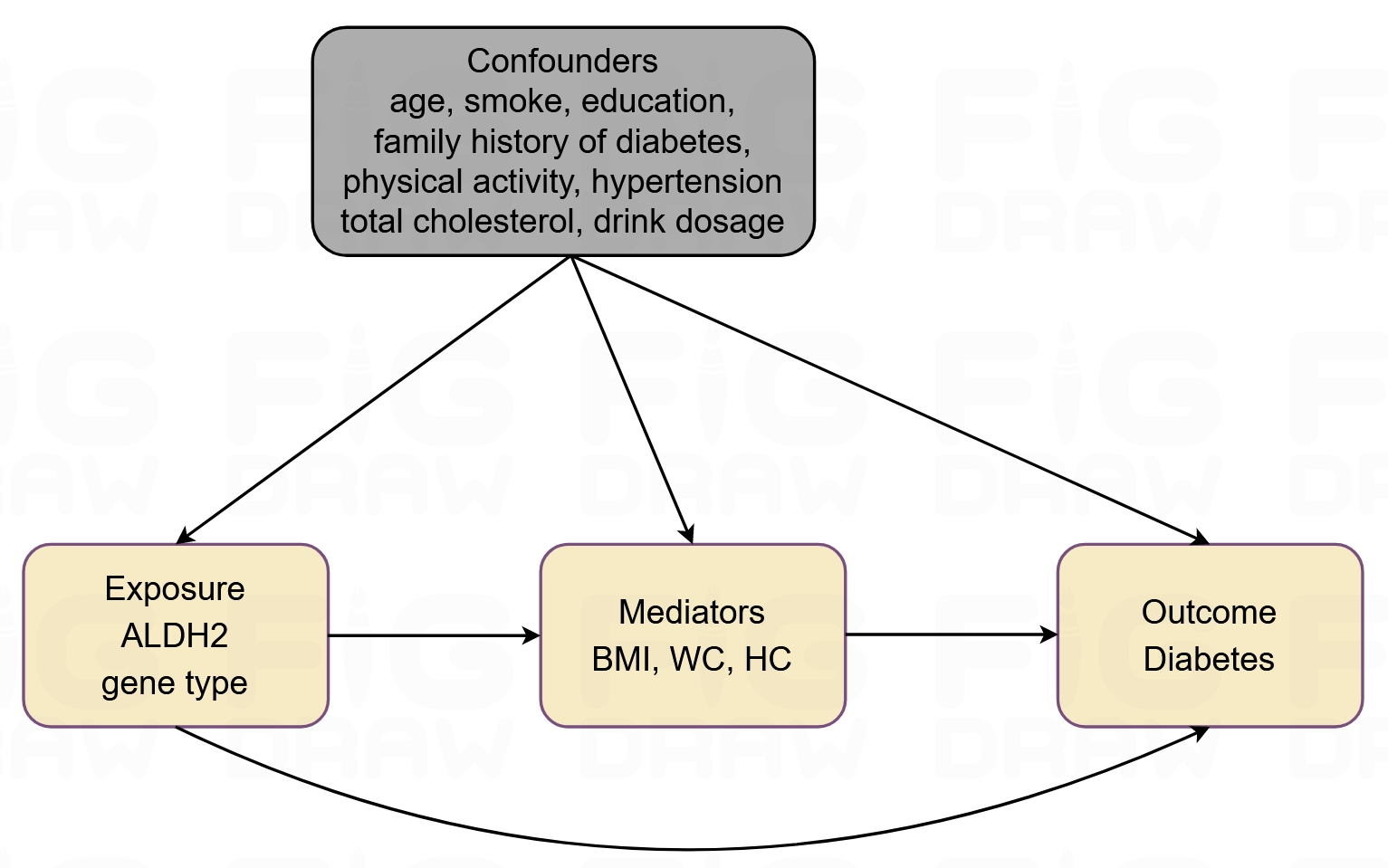

Supplement: Supplementary Figure 1 — The DAG depicting the relationship between confounders, exposure, mediators and outcome. [file Image1.jpeg]

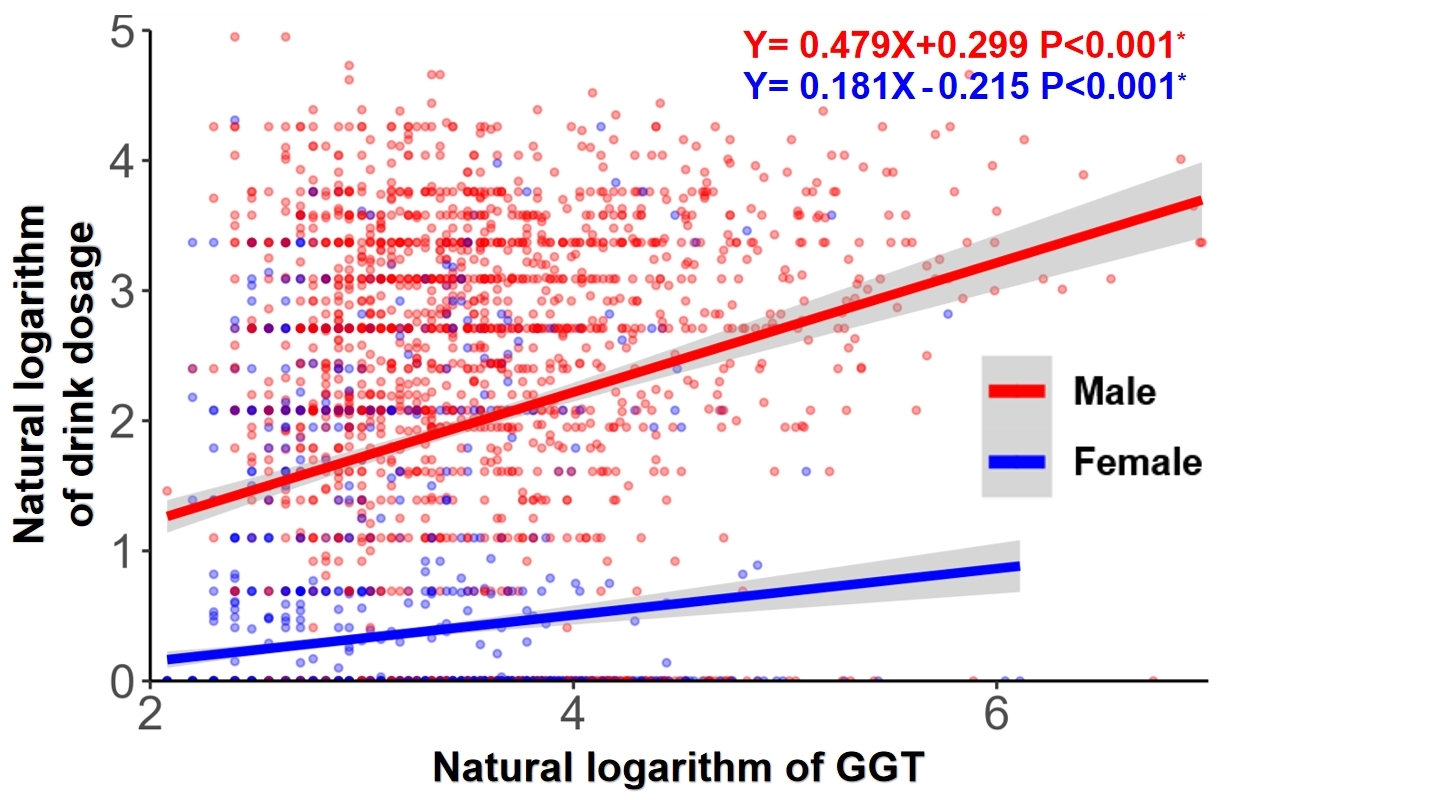

Supplement: Supplementary Figure 2 — Association between γ-glutamyltransferase and drinking dosage. [file Image2.jpeg]
