# Supplementary material for: Understanding the link between ALDH2 genotypes and diabetes
Source: Front Endocrinol (Lausanne). 2025 Feb 19;16:1451722. doi: 10.3389/fendo.2025.1451722 (PMC11879816; doi:10.3389/fendo.2025.1451722)
Supplement: Supplementary file 3 [file Table1.doc]

Table S1. Combined outcomes of all participants

| **Outcomes^a^** | Data presentation | | | | P-value^f^ |
| --- | --- | --- | --- | --- | --- |
|  | GG (N=3160)^a^ | GA/AA (N=1375)^a^ | GA (N=1249)^a^ | AA (N=126)^a^ |  |
| Age^b^ (year) | 54.83±8.74 | 55.04±8.92 | 55.14±8.94 | 54.05±8.73 | 0.470 |
| Gender (M/F) | 1597/1563 | 675/700 | 630/619 | 45/81 | 0.370 |
| BMI^b^ (kg/m^2^) | 23.4±3.24 | 23.12±3.18 | 23.13±3.18 | 22.96±3.21 | 0.006* |
| Education (uneducated/primary/ junior/senior and above) | 1422/1169/404/75 | 661/492/176/17 | 593/459/154/17 | 69/35/16/3 | 0.079 |
| Family history of diabetes^c^ (n, %) | 148 (4.7) | 57 (4.1) | 52 (4.2) | 5 (4.0) | 0.774 |
| Physical activity^c^ (n, %) | 670 (26.8) | 290 (26.8) | 263 (26.8) | 27 (26.5) | 0.955 |
| Smoker^c^ (n, %) | 548 (18.5) | 224 (17.1) | 206 (17.3) | 18 (15.1) | 0.279 |
| Drinker^c^ (n, %) | 1482 (49.3) | 361 (27.1) | 355 (29.3) | 6 (4.9) | <0.001* |
| Drink dosage^d^ (g/d) | 0 (0-44) | 0 (0-4.71) | 0 (0-7.86) | 0 (0-0) | / |
| Ln drink dosage^e^ | 1.85±2.01 | 0.96±1.67 | 1.04±1.71 | 0.19±0.83 | <0.001* |
| T2DM^c^ (n, %) | 538 (17.0) | 182 (13.2) | 161 (12.9) | 21 (16.7) | 0.001* |
| FPG^d^ (mmol/L) | 5.73(5.34-6.23) | 5.67(5.28-6.10) | 5.66 (5.27-6.1) | 5.79 (5.34-6.22) | / |
| Ln FPG^e^ | 1.78±0.19 | 1.76±0.17 | 1.76±0.17 | 1.78±0.19 | 0.001* |
| P2hPG^d^ (mmol/L) | 6.96(5.8-8.63) | 6.71(5.71-8.11) | 6.7 (5.68-8.08) | 6.98 (6.02-8.67) | / |
| Ln P2hPG^e^ | 1.98±0.37 | 1.94±0.35 | 1.94±0.35 | 2.01±0.36 | <0.001* |
| HbA1c^d^ (%) | 5.6(5.3-5.9) | 5.6(5.3-5.9) | 5.6 (5.3-5.9) | 5.6 (5.3-5.9) | / |
| Ln HbA1c^e^ | 1.73±0.13 | 1.73±0.12 | 1.73±0.11 | 1.74±0.13 | 0.914 |
| HOMA-IR^d^ | 1.44 (0.99-2.12) | 1.38 (0.94-2.14) | 1.38 (0.94-2.13) | 1.44 (0.92-2.18) | / |
| Ln HOMA-IR^e^ | 0.38±0.64 | 0.34±0.65 | 0.34±0.65 | 0.38±0.71 | 0.089 |
| HOMA-β^d^ | 48.50 (33.07-71.66) | 50.39 (33.91-71.77) | 50.64 (34.08-72.31) | 46.48 (31.11-64.75) | / |
| Ln HOMA-β^e^ | 3.87±0.63 | 3.90±0.61 | 3.90±0.61 | 3.86±0.64 | 0.150 |
| Hypertension^c^ (n, %) | 1494 (47.3) | 630 (45.8) | 567 (45.4) | 63 (50.0) | 0.365 |
| LDL-C^b^ (mmol/L) | 2.98±0.84 | 2.97±0.82 | 2.96±0.81 | 3.14±0.87 | 0.790 |
| HDL-C^b^ (mmol/L) | 1.72±0.44 | 1.70±0.40 | 1.69±0.41 | 1.71±0.38 | 0.088 |
| ^a^All the definitions were same as Table 1. ^b^Normally distributed variables are presented as the mean±standard error. ^c^Categorical variables are presented as numbers (percentages). ^d^Non-normally distributed variables are presented as the median (interquartile range). ^e^Non-normally distributed variables were naturally log-transformed. ^f^P-values were calculated from GA/AA vs. GG, and they were from χ^2^ test for categorical variables, from Student’s t-test for normally distributed variable, and Mann-Whitney U test for non-normally distributed variables. *P≤0.05 | | | | | |
