# Supplementary material for: Understanding the link between ALDH2 genotypes and diabetes
Source: Front Endocrinol (Lausanne). 2025 Feb 19;16:1451722. doi: 10.3389/fendo.2025.1451722 (PMC11879816; doi:10.3389/fendo.2025.1451722)
Supplement: Supplementary file 4 [file Table2.docx]

Table S2. Mediation analysis and sensitivity analysis for association between ALDH2 genotype and diabetes in male participants

| Mediation analysis  Male Diabetes | | Direct Effect^a^ (OR) | Indirect Effect^b^ (OR) | Total Effect (OR) | Proportion Mediated (%) | P-value |
| --- | --- | --- | --- | --- | --- | --- |
| **Without imputed drinking dosage data** | | | | | | |
| BMI | 95% CI | 0.769 [0.594, 0.971] | 0.935 [0.892, 0.979] | 0.719 [0.554, 0.920] | 17.9 [4.4, 73.0] | 0.01* |
|  | E-value | 1.924 | 1.343 | 2.127 |  |  |
| Waist Circumference | 95% CI | 0.799 [0.630, 0.981] | 0.897 [0.837, 0.949] | 0.716 [0.558, 0.888] | 29.1 [12.0, 86.3] | 0.01* |
|  | E-value | 1.813 | 1.474 | 2.140 |  |  |
| Hip Circumference | 95% CI | 0.740 [0.551, 0.901] | 0.966 [0.930, 0.994] | 0.714 [0.546, 0.893] | 8.9 [1.1, 25.0] | 0.02* |
|  | E-value | 2.042 | 1.227 | 2.148 |  |  |
| BMI+WC+HC | 95% CI | 0.775 [0.584, 0.970] | 0.902 [0.842, 0.940] | 0.699 [0.511, 0.859] | 25.4 [13.1, 78.2] | 0.01* |
|  | E-value | 1.883 | 1.460 | 2.199 |  |  |
| Drink dosage^a^ | 95% CI | 0.707 [0.557, 0.911] | 1.011 [0.845, 1.122] | 0.715 [0.543, 0.858] | -2.7 [-43.7, 55.3] | 0.86 |
|  | E-value | 2.182 | 1.118 | 2.147 |  |  |
| **With imputed drinking dosage data** | | | | | | |
| BMI | 95% CI | 0.781 [0.573, 0.992] | 0.935 [0.880, 0.977] | 0.730 [0.539, 0.927] | 18.9 [4.8, 75.4] | 0.01* |
|  | E-value | 1.870 | 1.344 | 2.082 |  |  |
| Waist Circumference | 95% CI | 0.809 [0.631, 1.055] | 0.897 [0.834, 0.951] | 0.726 [0.57, 0.924] | 30.4 [10.0, 127.0] | 0.01* |
|  | E-value | 1.776 | 1.472 | 2.099 |  |  |
| Hip Circumference | 95% CI | 0.753 [0.566, 0.966] | 0.961 [0.927, 0.994] | 0.724 [0.555, 0.934] | 10.6 [1.2, 47.1] | 0.02* |
|  | E-value | 1.987 | 1.245 | 2.106 |  |  |
| BMI+WC+HC | 95% CI | 0.780 [0.579, 0.971] | 0.901 [0.844, 0.962] | 0.702 [0.521, 0.865] | 26.0 [6.6, 76.2] | 0.01* |
|  | E-value | 1.884 | 1.460 | 2.200 |  |  |
| Drink dosage^a^ | 95% CI | 0.704 [0.507, 0.883] | 1.027 [0.892, 1.200] | 0.723 [0.558, 0.873] | -6.8 [-51.7, 29.1] | 0.56 |
|  | E-value | 2.198 | 1.199 | 2.112 |  |  |
| Abbreviation: BMI=body mass index; OR=odds ratio. ^a^Direct Effect is the pure natural direct effect calculated by CMAverse R package. ^b^Indirect Effect is the total natural indirect effect calculated by CMAverse R package. All outcomes were adjusted by age, smoke. education, exercise, family history of diabetes, LDL-C, HDL-C, hypertension and drink dosage. GG was regarded as control group, and GA/AA was regarded as exposure group. ^a^Drink dosage was not adjusted during the analysis of drink dosage. *P≤0.05. | | | | | | |
