# Supplementary material for: Understanding the link between ALDH2 genotypes and diabetes
Source: Front Endocrinol (Lausanne). 2025 Feb 19;16:1451722. doi: 10.3389/fendo.2025.1451722 (PMC11879816; doi:10.3389/fendo.2025.1451722)
Supplement: Supplementary file 5 [file Table3.docx]

Table S3. Estimates of association between ALDH2 rs671 genotype and alcohol consumption

| Alcohol consumption measure | Data Imputation | Cases/  individuals | Effect estimate(95%) | | | P valueh |
| --- | --- | --- | --- | --- | --- | --- |
|  |  |  | GG | GA | AA |  |
| **Log transformed dataa** |  |  |  | % difference | |  |
| number of participants |  | NA/4536 | 3161 | 1249 | 126 |  |
| intake volumeb (units/week) | Before | NA/3670 | ref. | -50.56% (-55.44%, -45.15%) | -75.93% (-85.71%, -73.12%) | <0.001* |
|  | After | NA/4536 | ref. | -44.63% (-49.44%, -39.37%) | -69.52% (-76.19%, 60.98%) | <0.001* |
| γ-glutamyltransferase (U/L) | Before | NA/4522 | ref. | -15.21% (-18.94%, -11.32%) | -23.00% (-31.85%, -12.99%) | <0.001* |
|  | After | NA/4522 | ref. | -15.21% (-18.94%, -11.32%) | -23.00% (-31.85%, -12.99%) | <0.001* |
| **Male** |  |  |  |  |  |  |
| number of participants |  | NA/2273 | 1597 | 614 | 62 |  |
| intake volumec (units/week) | Before | NA/2272 | ref. | -58.40% (-63.44%, -52.67%) | -88.54% (-92.31%, -82.91%) | <0.001* |
|  | After | NA/2273 | ref. | -58.48% (-63.39%, -52.90%) | -83.89% (-89.25%, -75.86%) | <0.001* |
| **Female** |  |  |  |  |  |  |
| number of participants |  | NA/2263 | 1563 | 636 | 64 |  |
| intake volumed (units/week) | Before | NA/2263 | ref. | -30.42% (-36.11%, -24.23%) | -39.04% (-50.06%, -25.60%) | <0.001* |
|  | After | NA/2263 | ref. | -25.59% (-30.86%, -19.92%) | -33.98% (-44.64%, -21.27%) | <0.001* |
| **Drinker** |  |  | GG | GA and AA^f^ | |  |
| number of participants |  | NA/2034 | 1634 | 400 | |  |
| intake volumee (units/week) | Before | NA/1843 | ref. | -18.53% (-26.79%, -9.33%) | | <0.001* |
|  | After | NA/2034 | ref. | -18.86% (-27.06%, -9.75%) | | <0.001* |
| **Categorical data** |  |  |  | Odd ratio | |  |
| number of participants |  | 1821/4526 | 1053 | 664 | 104 |  |
| lifelong abstainerg | Before | 1821/4526 | ref. | 2.261 (1.979, 2.585) | 9.419 (5.911, 15.008) | <0.001* |
|  | After | 1821/4526 | ref. | 2.261 (1.979, 2.585) | 9.419 (5.911, 15.008) | <0.001* |
| NA=not applicable. ^a^Non-normally distributed variable were natural log transformed and mean differences on the log scale were converted to percentage differences. ^b^Alcohol units in Chinese units; 1 CN unit approximates 22.05g ethanol. ^c^For males only. ^d^For females only. ^e^For drinkers only (current drinkers and former drinkers). ^f^As there were only 8 individuals with the genotype of AA, mean difference was estimated between rs671 A-allele non-carriers and carriers. ^g^lifelong abstainer are those who declare never drink alcohol. ^h^P value derived from one-way ANOVA. ^*^P≤0.05 | | | | | | |
