# Supplementary material for: Understanding the link between ALDH2 genotypes and diabetes
Source: Front Endocrinol (Lausanne). 2025 Feb 19;16:1451722. doi: 10.3389/fendo.2025.1451722 (PMC11879816; doi:10.3389/fendo.2025.1451722)
Supplement: Supplementary file 6 [file Table4.docx]

Table S4. Estimates for the effecct of the ALDH2 rs671 A-allele number on diabetes risk and related outcomes in male participants

| ALDH2 rs671 A-allele | Original Model | | | Adjusting Model 1^a^ | | Adjusting Model 2^b^ | | Adjusting Model 3^c^ | | Adjusting Model 4^d^ | |
| --- | --- | --- | --- | --- | --- | --- | --- | --- | --- | --- | --- |
|  | OR/MD^f^ [95% CI] | P | OR/MD^f^ [95% CI] | | P | OR/MD^f^ [95% CI] | P | OR/MD^f^ [95% CI] | P | OR/MD^f^ [95% CI] | P |
| ***Multiple linear/logistic regression*** | | | | | | | | | | | |
| T2DM | 0.729  [0.579, 0.919] | 0.007* | 0.733  [0.577, 0.931] | | 0.011 | 0.736  [0.579, 0.937] | 0.013 | 0.800  [0.621, 1.030] | 0.084 | 0.823  [0.637, 1.063] | 0.136 |
| FPG^e^ | -1.62%  [-2.52%, -0.73%] | <0.001* | -1.57%  [-2.46%, -0.67%] | | <0.001* | -1.51%  [-2.41%, -0.61%] | <0.001* | -1.18%  [-2.07%, -0.28%] | 0.010* | -0.84%  [-1.73%, 0.06%] | 0.062 |
| P2hPG^e^ | -2.74%  [-4.32%, -1.17%] | 0.001* | -2.69%  [-4.27%, -1.12%] | | 0.001* | -2.59%  [-4.17%, -1.02%] | 0.002* | -1.37%  [-3.05%, 0.30%] | 0.112 | -0.86%  [-2.54%, 0.81%] | 0.325 |
| HbA1c^e^ | 0.12%  [-0.46%, 0.70%] | 0.774 | 0.17%  [-0.41%, 0.75%] | | 0.594 | 0.17%  [-0.41%, 0.75%] | 0.576 | 0.12%  [-0.58%, 0.81%] | 0.728 | 0.29%  [-0.41%, 0.98%] | 0.362 |
| HOMA-IR^e^ | -40.24%  [-61.14%, -19.33%] | <0.001* | -41.81%  [-63.51%, -20.12%] | | <0.001* | -44.18%  [-63.51%, -24.85%] | <0.001* | -44.97%  [-65.09%, -24.85%] | <0.001* | -15.78%  [-33.53%, 1.97%] | 0.136 |
| HOMA-beta^e^ | -0.30%  [-1.67%, 1.08%] | 0.684 | -0.24%  [-1.56%, 1.08%] | | 0.729 | -0.46%  [-1.72%, 0.81%] | 0.477 | -0.94%  [-2.31%, 0.43%] | 0.171 | -0.19%  [-1.51%, 1.13%] | 0.786 |
| BMI | -0.598  [-0.857, -0.339] | <0.001* | -0.554  [-0.809, -0.299] | | <0.001* | -0.582  [-0.811, -0.353] | <0.001* | -0.394  [-0.635, -0.153] | 0.001* | / | / |
| Waist Circumference | -2.401  [-3.165, -1.637] | <0.001* | -2.373  [-3.126, -1.62] | | <0.001* | -2.450  [-3.118, -1.782] | <0.001* | -1.656  [-2.358, -0.954] | <0.001* | / | / |
| Hip Circumference | -0.992  [-1.517, -0.467] | <0.001* | -0.961  [-1.488, -0.434] | | <0.001* | -0.994  [-1.488, -0.500] | <0.001* | -0.781  [-1.304, -0.258] | 0.003* | / | / |
| Abbreviation: BMI=body mass index; OR=odds ratio; MD=mean difference; CI=confidence interval. ^a^Model 1 include age, smoke, education, physical activity, family history of diabetes. ^b^Model 2=Model 1+LDL-C+HDL-C +hypertension. ^c^Model 3 (full model)=Model 2+drink dosage. ^d^Model 4 (sensitivity model)=Model 2+BMI+waist circumference. ^e^Non-normally distributed variable were natural log transformed and resulted in percentage differences. ^f^ALDH2 rs671 A-allele number was regarded as the exposure variable in the regression analyses. *P≤0.05. | | | | | | | | | | | |
