# Supplementary material for: Understanding the link between ALDH2 genotypes and diabetes
Source: Front Endocrinol (Lausanne). 2025 Feb 19;16:1451722. doi: 10.3389/fendo.2025.1451722 (PMC11879816; doi:10.3389/fendo.2025.1451722)
Supplement: Supplementary file 7 [file Table5.docx]

Table S5. Mediating effect of beta cell function and insulin resistance on the association between ALDH2 genotype and diabetes in male participants

| Male Diabetes | | Direct Effect (OR) | Indirect Effect (OR) | Total Effect (OR) | Proportion Mediated (%) | P value^b^ |
| --- | --- | --- | --- | --- | --- | --- |
| **HOMA-IR** | |  |  |  |  |  |
| Adjusting Model 1^a^ | 95% CI | 0.807  [0.656, 0.988] | 0.895  [0.815, 0.969] | 0.722  [0.571, 0.925] | 30.5  [9.9, 92.3] | 0.02^*^ |
|  | E value^b^ | 1.785 | 1.480 | 2.117 |  |  |
| Adjusting Model 2^a^ | 95% CI | 0.793  [0.616, 1.007] | 0.982  [0.897, 1.058] | 0.778  [0.586, 0.998] | 6.6  [-53.7, 54.0] | 0.57 |
|  | E value^b^ | 1.835 | 1.157 | 1.889 |  |  |
| **HOMA-beta** | |  |  |  |  |  |
| Adjusting Model 1^a^ | 95% CI | 0.741  [0.608, 0.942] | 0.986  [0.954, 1.020] | 0.730  [0.579, 0.929] | 4.0  [-7.6, 24.6] | 0.45 |
|  | E value^b^ | 2.037 | 1.137 | 2.081 |  |  |
| Adjusting Model 2^a^ | 95% CI | 0.758  [0.600, 0.994] | 1.012  [0.986, 1.064] | 0.767  [0.603, 1.010] | -4.0  [-114.6, 11.3] | 0.48 |
|  | E value^b^ | 1.884 | 1.460 | 2.200 |  |  |
| ^a^Model 1 include age, smoke, education, physical activity, family history of diabetes, LDL-C, HDL-C and hypertension. Model 2=Model 1+BMI+WC. ^b^Both P value and E value derived from the CMAverse R package. ^*^P≤0.05 | | | | | | |
